# Minimally Invasive Retrieval of Long-Separated Endodontic Instruments Using a Nitinol Loop Device: A Report of Two Cases

**DOI:** 10.7759/cureus.92978

**Published:** 2025-09-22

**Authors:** Majed Amran, Aziz Abdullah

**Affiliations:** 1 Department of Endodontics and Operative Dentistry, Tishreen University, Lattakia, SYR; 2 Department of Conservative Dentistry and Endodontics, Tishreen University, Lattakia, SYR

**Keywords:** dental operating microscope (dom), endodontic, nitinol loop device, root canal therapy, separated instruments

## Abstract

Separation of endodontic instruments during root canal treatment is a well-recognized complication that can significantly compromise the prognosis of the affected tooth. Managing such incidents requires careful clinical judgment and technique selection to retrieve or bypass the fragment while minimizing damage to the canal system. This report presents two cases involving the management of long-separated nickel-titanium rotary instruments, one of which extended approximately 3 mm beyond the apical foramen.

## Introduction

Endodontic treatment involves the meticulous cleaning, shaping, and obturation of the root canal system to eliminate infection and preserve the natural tooth [1]. Despite significant advances in endodontic techniques and instrument design, procedural complications can still arise. One of the most challenging and frequently encountered complications is the separation, or breakage, of endodontic instruments within the root canal [2].

Instrument separation is a multifactorial problem often associated with anatomical complexities such as curved or calcified canals, as well as mechanical factors, including metal fatigue, excessive force, and improper technique. While the presence of a broken instrument does not always guarantee treatment failure, it significantly complicates the clinical procedure and may reduce the long-term success rate if not managed appropriately [3].

The occurrence of broken instruments ranges between 2% and 6% and presents both clinical and psychological challenges for practitioners. Successful management requires a thorough understanding of the causes, prevention strategies, and available techniques for retrieval or bypassing of the separated fragment [4].

A thorough understanding of the causes, risk factors, and management strategies related to instrument separation is essential for endodontists and general dental practitioners. With appropriate prevention measures, careful technique, and the use of modern retrieval systems, clinicians can minimize the risks and effectively handle such complications when they arise [5].

The selection of a device depends on factors such as the location, size, and type of the fractured instrument. Effective use of these tools can significantly increase the chances of successful instrument removal while preserving as much tooth structure as possible, ultimately contributing to the long-term success of the endodontic procedure. Several specialized devices have been developed to facilitate the retrieval or bypassing of these fragments within the root canal system [6]. These include ultrasonic tips, micro-tube systems like the Masserann kit, and Dental Broken Root Canal Pen Remover Endo (ELIJAH, Zhengzhou, China).

The Dental Broken Root Canal Pen Remover Endo is an innovative retrieval system that employs a nitinol wire loop to grasp and extract separated instruments. Below are two cases demonstrating the successful removal of fractured instruments located in extraradicular positions using this looping device.

## Case presentation

Case 1

**Figure 1:**
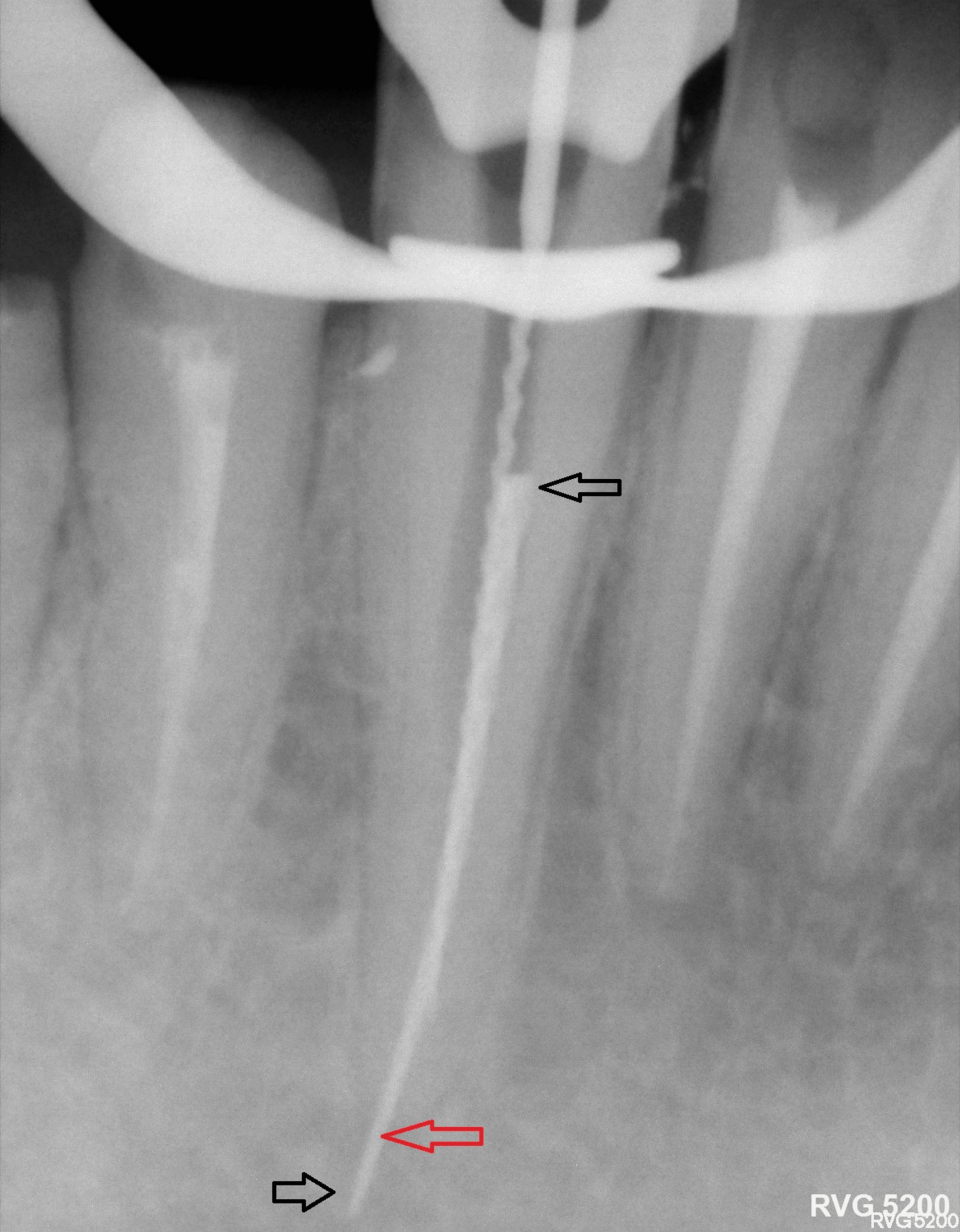
A separated endodontic instrument is lodged within the middle third of the root canal of tooth #43, extending through the canal and protruding beyond the apical foramen. The separated instrument fragment extends from the area marked by the two black arrows, originating in the middle third of the root canal, and overextended beyond the apical foramen of tooth #43, which is indicated by the red arrow.

A 36-year-old male patient was referred by his general dentist for the management of a separated endodontic instrument in tooth #43. The patient reported localized pain following the initiation of root canal treatment. Intraoral examination revealed no signs of swelling or sinus tract formation. A periapical radiograph demonstrated a separated instrument extending from the middle third of the root canal and overextended beyond the apical foramen (Figure 1).

**Figure 2:**
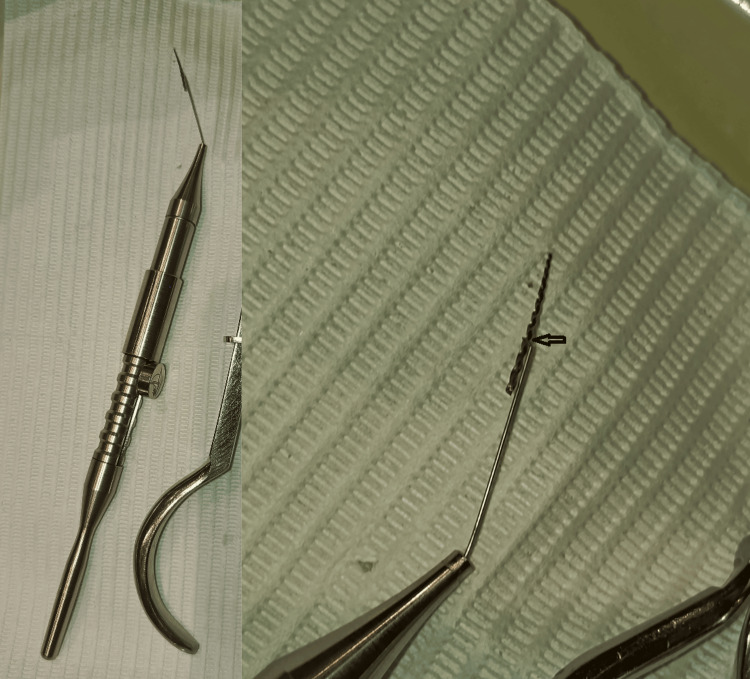
The image illustrates the separated file fragment, which was successfully removed using the Dental Broken Root Canal Pen Remover Endo device. The black arrows indicate the location where the loop of the Dental Broken Root Canal Pen Remover Endo device encircles and tightens around the separated instrument fragment for its removal.

**Figure 3:**
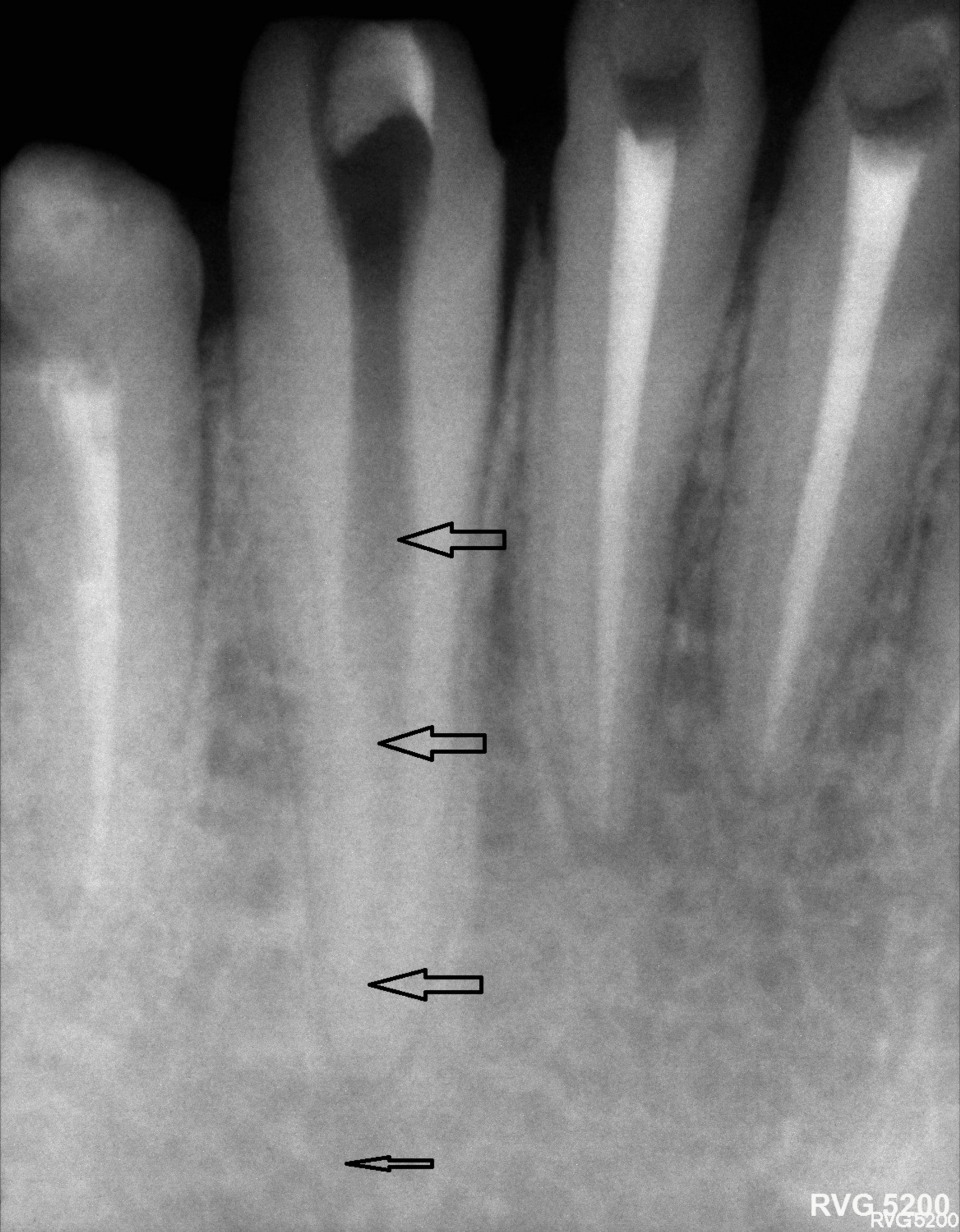
The image shows the root canal of tooth #43 after the successful removal of the separated instrument. The black arrows indicate the previous location of the separated instrument fragment within the root canal of tooth #43, now clearly visible and unobstructed following successful removal.

**Figure 4:**
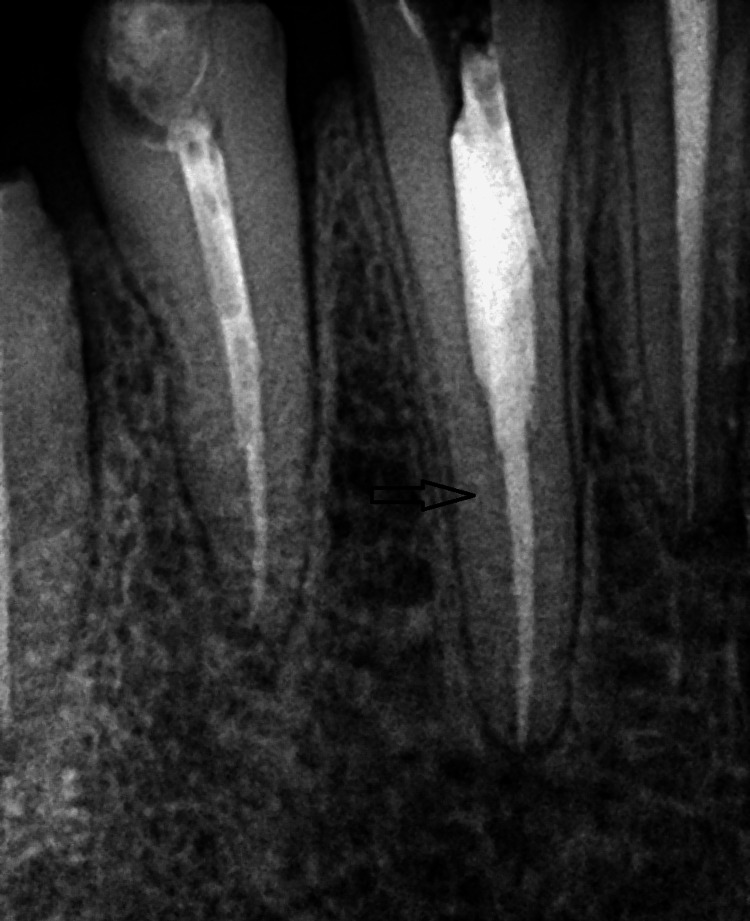
The image shows the root canal of tooth #43 after removal of the separated file, now fully obturated and sealed. The image shows the root canal of tooth #43 after successful removal of the separated instrument fragment using the Dental Broken Root Canal Pen Remover Endo device. The black arrows indicate the previous location of the separated instrument within the canal, which is now clearly visible and unobstructed. Following removal, the canal has been thoroughly cleaned, shaped, and fully obturated, as highlighted by the black arrow pointing to the obturated canal. The root canal appears well sealed with no signs of perforation or damage, indicating favorable conditions for the continuation of endodontic treatment.

The patient was informed about the treatment plan, and written informed consent was obtained. The tooth was isolated using a rubber dam, and the canal was irrigated with sodium hypochlorite (NaOCl) under magnification with the aid of a dental operating microscope. Under magnification, the coronal portion of the separated instrument was visualized within the canal. A Dental Broken Root Canal Pen Remover Endo device was introduced into the canal, positioning the nitinol loop around the coronal end of the fragment. The loop was then securely tightened to achieve firm engagement of the instrument in preparation for retrieval. The fractured instrument was successfully retrieved (Figures 2, 3), followed by obturation of the canal using gutta-percha and CeraSeal bioceramic sealer (Meta Biomed Co., Ltd., Cheongju-si, South Korea), with a combination of vertical compaction and warm injection techniques (Figure 4).

Case 2

**Figure 5:**
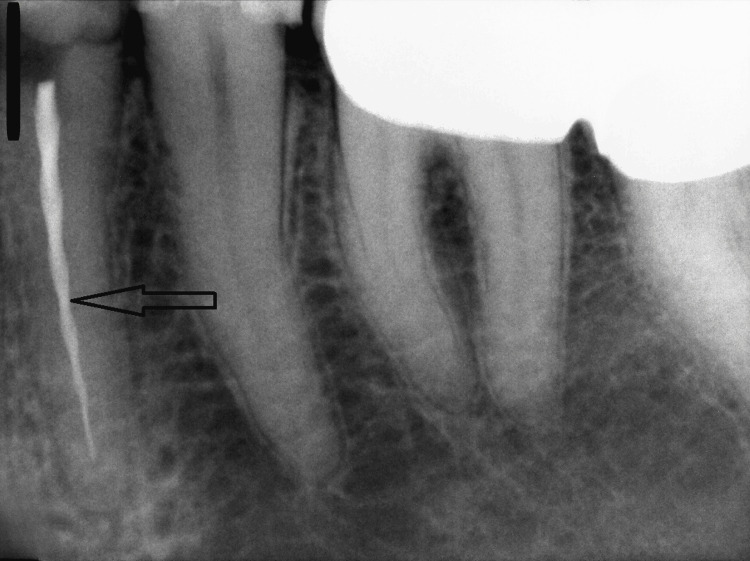
The image shows a separated file lodged within the root canal of tooth #34, with the black arrow indicating the exact location of the separated instrument.

A 45-year-old female patient was referred with a separated long file fragment lodged in tooth 34 (Figure 5).

**Figure 6:**
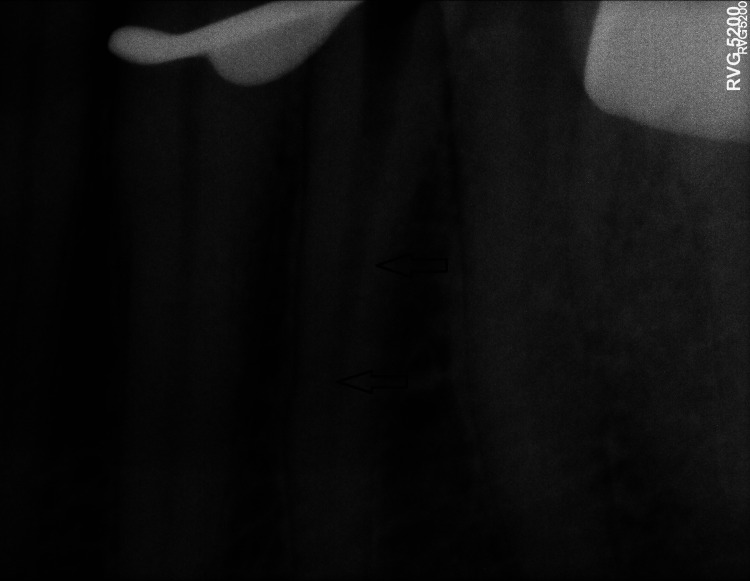
The image depicts the root canal of tooth #34 following the successful removal of a separated file. The two black arrows pinpoint the former location of the separated instrument within the canal, now clearly unobstructed. The canal appears clean and well-prepared, allowing for effective cleaning, shaping, and subsequent obturation.

Following clinical and radiographic evaluation, the fractured instrument was removed using a combined approach of ultrasonic instrumentation (Ultra X Ultrasonic Device, Eighteeth Co., Ltd., Changzhou, China) and the Dental Broken Root Canal Pen Remover Endo device. Initially, ultrasonic tips were carefully applied around the coronal portion of the separated file to selectively remove dentin and loosen the fragment without damaging the canal walls. Once sufficient space and mobility were achieved, the Root Canal Pen Remover Endo was used to securely engage and retrieve the separated file from the canal (Figure 6).

**Figure 7:**
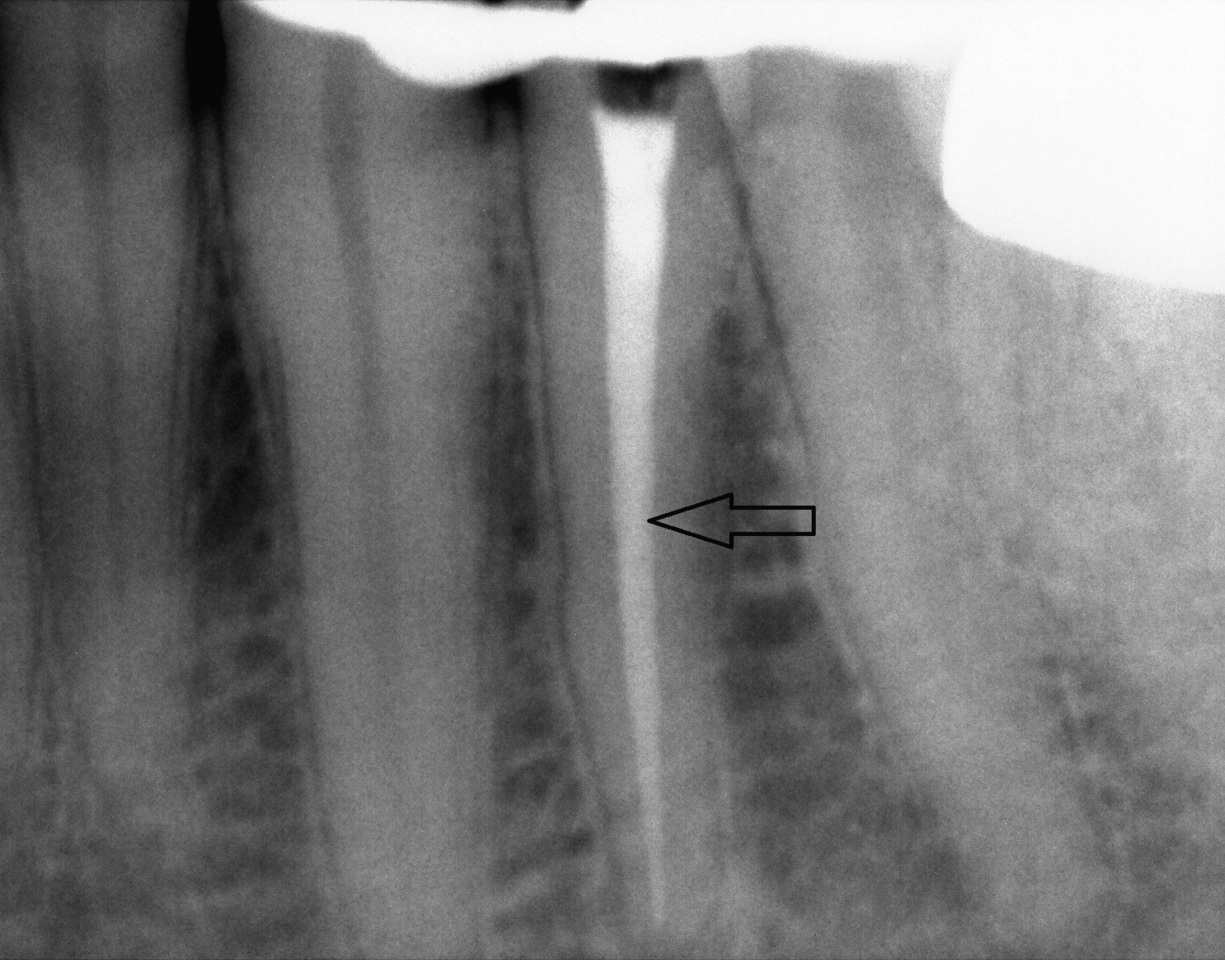
The image illustrates tooth #34 following the successful retrieval of the separated file from the root canal. The canal has been thoroughly cleaned, shaped, and subsequently obturated to ensure a hermetic seal. The black arrow points directly to the obturated canal, highlighting the dense and continuous filling material that extends to the apex, indicating an effective and complete root canal treatment.

This combined approach allowed for controlled removal of the fragment while preserving the structural integrity of the tooth. The procedure was completed without complications, enhancing the prognosis of the subsequent endodontic treatment. The tooth was then obturated using gutta-percha and CeraSeal bioceramic sealer (Meta Biomed Co., Ltd.), employing a combination of vertical compaction and warm injection techniques (Figure 7).

## Discussion

Separation of endodontic instruments remains one of the most challenging complications encountered during root canal treatment. The presence of a fractured instrument within the canal can impede thorough cleaning and shaping, potentially compromising the disinfection process and long-term treatment success. The incidence of instrument separation varies but is reported to range from 2% to 6%, with nickel-titanium rotary files being particularly susceptible due to their mechanical properties and the stress imposed during canal instrumentation [7].

Successful management of separated instruments depends largely on their location, size, and accessibility, as well as the clinician’s ability to balance effective retrieval with preservation of root canal anatomy. Instruments fractured in the coronal or middle thirds of the canal are generally more amenable to removal, whereas those located in the apical third or extending beyond the apex pose greater difficulty and risk. In the cases presented, the fractured instruments were located in positions that allowed for the use of ultrasonic techniques combined with a specialized retrieval device, the Dental Broken Root Canal Pen Remover Endo, which employs a nitinol wire loop to securely engage and extract the fragment [8].

Ultrasonic instrumentation plays a pivotal role in creating space around the fragment by selectively removing dentin and loosening the broken file without causing excessive damage to the canal walls. This conservative approach minimizes the risk of root weakening or perforation. Once the fragment is sufficiently mobilized, the nitinol loop of the pen remover device provides a secure grip, enabling controlled retrieval under magnification. The synergy of these techniques in the presented cases facilitated effective fragment removal while preserving tooth structure and ensuring canal patency [9].

The preservation of tooth integrity is critical, as aggressive dentin removal can predispose teeth to fracture and jeopardize their prognosis. The use of magnification and illumination through dental operating microscopes enhances visualization, thereby improving the precision of retrieval procedures and minimizing iatrogenic damage [10].

While some studies suggest that retained apical fragments may not necessarily compromise treatment outcomes if adequate disinfection is achieved, the preferred clinical objective remains the complete removal or bypass of the obstruction to enable thorough chemomechanical preparation [11]. In both cases described, successful fragment retrieval allowed for subsequent obturation with gutta-percha and bioceramic sealer, aiming to optimize the long-term prognosis.

Limitations of this case report are that the cases involved fragments located in accessible canal regions; more complex scenarios involving severely curved canals or deeply apical fragments may require alternative or adjunctive techniques. Further clinical studies with larger cohorts and extended follow-up are warranted to establish standardized protocols and compare the efficacy of different retrieval systems.

## Conclusions

This case report demonstrates the effective use of a modern retrieval system in conjunction with ultrasonic instrumentation and magnification for managing separated endodontic instruments. The combination facilitates minimally invasive removal, preserves tooth structure, and improves treatment prognosis. Nonetheless, further clinical studies with long-term follow-up are warranted to validate these findings across diverse clinical scenarios.
